# Fast single-photon detectors and real-time key distillation enable high secret-key-rate quantum key distribution systems

**DOI:** 10.1038/s41566-023-01168-2

**Published:** 2023-03-09

**Authors:** Fadri Grünenfelder, Alberto Boaron, Giovanni V. Resta, Matthieu Perrenoud, Davide Rusca, Claudio Barreiro, Raphaël Houlmann, Rebecka Sax, Lorenzo Stasi, Sylvain El-Khoury, Esther Hänggi, Nico Bosshard, Félix Bussières, Hugo Zbinden

**Affiliations:** 1 Group of Applied Physics, Genève, Switzerland; 2 ID Quantique SA, Acacias, Genève, Switzerland; 3 Lucerne School of Computer Science and Information Technology, Lucerne University of Applied Sciences and Arts, Rotkreuz, Switzerland; 4 Present Address: University of Vigo, Vigo, Spain

**Keywords:** Quantum information, Quantum optics

## Abstract

Quantum key distribution has emerged as the most viable scheme to guarantee information security in the presence of large-scale quantum computers and, thanks to the continuous progress made in the past 20 years, it is now commercially available. However, the secret key rates remain limited to just over 10 Mbps due to several bottlenecks on the receiver side. Here we present a custom multipixel superconducting nanowire single-photon detector that is designed to guarantee high count rates and precise timing discrimination. Leveraging the performance of the detector and coupling it to fast acquisition and real-time key distillation electronics, we remove two major roadblocks and achieve a considerable increase of the secret key rates with respect to the state of the art. In combination with a simple 2.5-GHz clocked time-bin quantum key distribution system, we can generate secret keys at a rate of 64 Mbps over a distance of 10.0 km and at a rate of 3.0 Mbps over a distance of 102.4 km with real-time key distillation.

## Abstract (web summary)

In combination with a 2.5-GHz clocked time-bin quantum key distribution system, secret keys are generated at a rate of 64 Mbps over a distance of 10.0 km and at a rate of 3.0 Mbps over a distance of 102.4 km with real-time key distillation.

## Main

Quantum key distribution (QKD) allows the exchange of cryptographic keys at a distance without assumptions on the technological limits of a possible eavesdropper, in particular their computational power^1,2^. In contrast, currently used public key systems rely on computationally demanding tasks^3,4^. Although, nowadays, an eavesdropper is bound to use classical computers, this could change in the near future with the advent of large-scale quantum computers, which would render an eavesdropper able to use powerful attacks that today’s public key systems cannot withstand^5^. The security of QKD, however, is solely based on the laws of quantum mechanics, so, together with the one-time pad^6^, private communication can be ensured even in a future where quantum computers are widely available.

Since the advent of the QKD era with the BB84 protocol^1^, a variety of other protocols have been developed^2,7–9^. Although the complexity and level of device independence differ among protocols, the main goals remain the same, namely to increase the distance over which a secret key can be generated or, conversely, to maximize the secret key rate (SKR) over a certain distance. To give some context, we consider the use-case of encrypted video conferencing. The United States Federal Communications Commission recommends a download rate of 6 Mbps for this application^10^, so, with one-time-pad encryption, one needs an SKR equal to this rate per user. For more demanding applications, such as data centres, much higher SKRs can be required. Recently, it was demonstrated that a single QKD link can achieve a sustainable SKR up to 13.72 Mbps over a channel equivalent to 10 km of single-mode fibre^11^. A proof-of-principle experiment using space division multiplexing showed that it would be possible to achieve an SKR of 105.7 Mbps over a distance of 7.9 km by using 37 QKD transmitters and receivers with a multicore fibre as the quantum channel^12^.

To increase the SKRs even further, without multiplexing, a QKD system needs to fulfil a few key requirements. In the first place, the transmitter must emit qubits at a high repetition rate. However, a high repetition rate is only useful, in particular at shorter distances, if (1) the single-photon detectors are able to count at high rates with high efficiency and low timing jitter, (2) the readout and sifting electronics are able to process these rates and (3) the post-processing unit is capable of correcting the key (with low leakage) and performing privacy amplification in real time.

In this Article we report on our efforts to improve these three factors. In particular, we present a custom superconducting nanowire single-photon detector (SNSPD) featuring high count rates and high efficiency. We discuss how to optimize the parameters of a QKD system for high SKR and demonstrate an implementation generating an SKR of more than 60 Mbps over 10 km and 3 Mbps over 100 km. We use a simplified BB84 with time-bin encoding and one decoy state clocked at 2.5 GHz (time bins of 100 ps separated by 100 ps)^13–15^, but the presented principles are valid also for polarization-based schemes^16^.

## Results

### Multi-pixel SNSPD

**Fig. 1:**
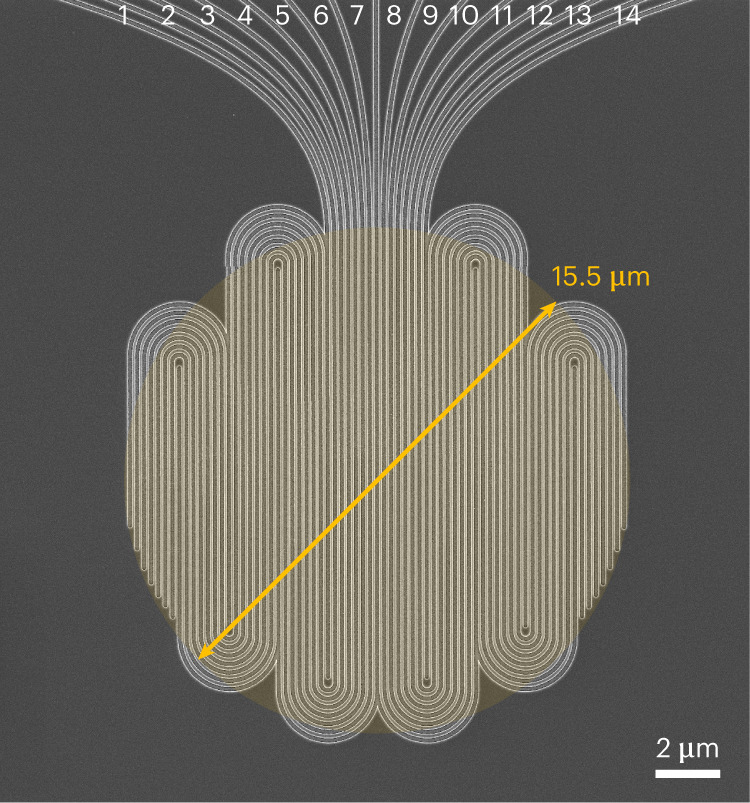
The SNSPD with 14 interleaved pixels. Image taken with a scanning electron microscope (SEM). The detector covers an area with a diameter of ~15.5 μm, which corresponds to an overlap of 99.7% (6*σ*) with the mode of the SMF-28 fibre. The width of the nanowires is 100 nm with a fill factor of 50%, and the interleaved design ensures uniform illumination of the pixels.

We designed the multipixel SNSPD^17,18^ such that high efficiency, low jitter and a high maximum count rate can be achieved simultaneously. We use niobium-titanium nitride (NbTiN), sputtered from a NbTi target in a nitrogen-rich atmosphere, as the superconducting material. The superconducting film has a thickness of ~9 nm and exhibits a critical temperature (*T*_c_) of 8.8 K. The detector is composed of 14 independent pixels arranged in an interleaved geometry (Fig. 1). The number of pixels was chosen to comply with the requirements of Bob, and the generated signals are amplified at 40 K with a custom-made amplifier board. Thanks to the large number of pixels and the interleaved design (which guarantees uniform illumination of the pixels), the probability that two detections occur during the recovery time on the same pixel is minimized^18^. The detector is integrated into an optical cavity designed to maximize photon absorption at 1,550 nm, and exhibits a maximum system detection efficiency (SDE) of 82% (Fig. 3). The detector covers the same area as a conventional single-pixel SNSPD (~200 μm^2^), so the length of each nanowire is greatly reduced, allowing for a much faster recovery time (on average <8 ns to be back at full efficiency). The fast recovery time of each pixel directly translates into the capability to reach ultrahigh detection rates when reading all 14 pixels simultaneously.

**Fig. 2:**
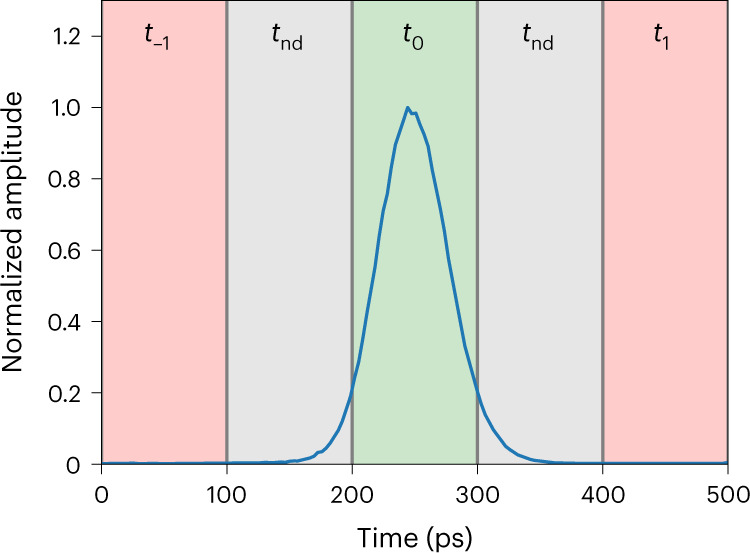
Timing resolution of detections. Histogram of the arrival times measured with one pixel (for a total count rate of 15.3 Mcps), which is the result of a convolution of the laser pulse shape and the jitter of the detector including readout electronics. Detections falling in the central green time bin *t*_0_ are correct, detections falling in the red time bins *t*_−1_ and *t*_1_ lead to errors, whereas events in the grey time bins *t*_nd_ are discarded to lower the QBER. In other words, jitter leads both to loss (*t*_nd_ bins) or errors (*t*_−1_ and *t*_1_ bins).

**Fig. 3:**
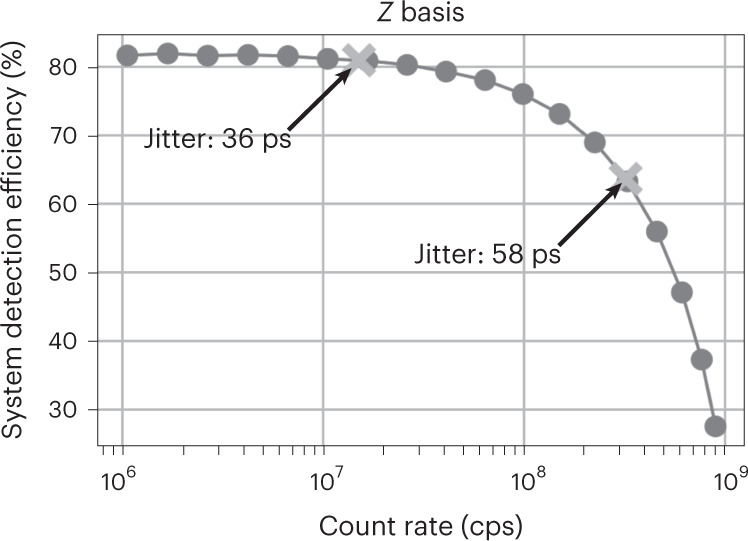
SDE of the multipixel detector versus count rate. At low count rates (below 10 Mcps) we measure a maximum SDE of 82%. As the count rate increases, the SDE begins to drop as more photons are reaching the detector within the dead time of each pixel. The crosses show the operating points for the performed key exchanges. At a distance of 10 km, the detector operates at 320 Mcps with SDE of 64% and temporal jitter of 58 ps, and at a distance of 100 km it operates at 15.0 Mcps with SDE of 81% and temporal jitter of 36 ps. The contributions to the jitter come both from the detector and the readout electronics.

We also characterized the jitter of each pixel at different bias currents (*I*_b_) and at a low count rate (100 kcps for each pixel), using a commercially available time-correlated single-photon-counting module. We obtained an average full-width at half-maximum (FWHM) jitter of 22 ps for bias currents (*I*_b_) close to the critical current (*I*_c_) and an average jitter of 26 ps for *I*_b_ = 0.9*I*_c_, which represents an excellent starting point for when the detector will be operated at high count rates. In fact, during the key exchange, Bob must measure the arrival time of the pulses and be sure to assign them to the correct time bin, which has a 100-ps duration (Fig. 2). At the high rates of the QKD system, more and more photon detections occur when the bias current in the SNSPD has not yet reached its maximum value, that is, before the current and efficiency have fully recovered, thus causing an increase of the jitter. One contribution to the jitter at high detection rates is the variation in the amplitude of the detection signal, and this contribution can be minimized by using constant fraction discriminators (CFDs) instead of threshold discriminators on Bob’s side. We designed and built CFDs optimized to be used with our multipixel detector, and with this readout electronics we simultaneously obtained a jitter below 60 ps and an efficiency of 64% at a count rate of 320 Mcps, which represents the operating point of the detector for our short-distance key exchange (Fig. 3).

It should be noted that, in the past, SPDs used in QKD experiments, such as avalanche photodiodes (APDs) and SNSPDs, have been shown to be vulnerable to hacking^19^. However, free-running multipixel SNSPDs with specifically designed readout electronics (including a shunt resistor to prevent latching) are particularly robust against such attacks^20^, and monitoring coincidence clicks between the pixels can be a powerful countermeasure^21^.

### Error correction and privacy amplification

**Fig. 4:**
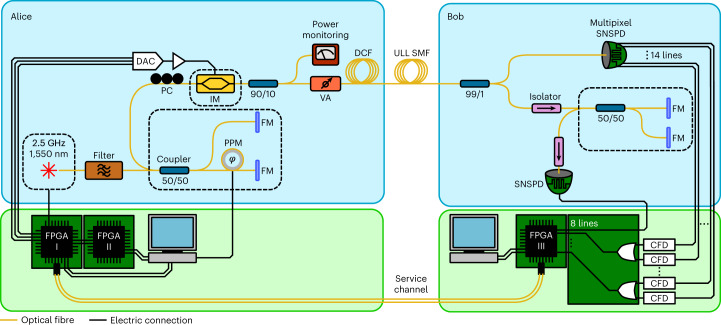
QKD set-up. Schematic representation of the QKD set-up with all the key components. CFD, constant fraction discriminator; DAC, digital-to-analogue converter; DCF, dispersion-compensating fibre; FM, Faraday mirror; FPGA, field-programmable gate array; IM, intensity modulator; PC, polarization controller; PPM, piezo-electric phase modulator; SNSPD, superconducting nanowire single-photon detector; ULL SMF, ultra-low-loss single-mode fibre; VA, variable attenuator. The dashed boxes are temperature-stabilized. FPGA I controls the state preparation, FPGA II is used for error correction, and FPGA III acquires the detection events. The sifting is done between FPGAs I and III. Both Michelson interferometers exhibit an imbalance of 200 ps.

The ultrafast SNSPD and post-processing error correction are implemented in our QKD system as shown in Fig. 4. For error correction, we use a quasi-cyclic low-density parity check code (LDPC) with a syndrome size of 1/6, which is implemented in field-programmable gate arrays (FPGAs)^22^. Bob calculates the syndrome and sends it to Alice. The resource-intensive error correction core runs on FPGA II (Xilinx Virtex-6 LXT; Fig. 4) on Alice’s side. One core can correct up to 110 Mcps and, by simply running two cores in parallel on the same FPGA, we achieve a throughput of 220 Mcps, which is high enough for our experiment. The privacy amplification is implemented on a consumer-type computer that receives the sifted key via a Generation 2 PCIe x4 connector (maximum throughput of 4 GB) from the FPGA. It runs on a consumer-type graphics processing unit (RTX 2070 Super Ventus OC) and has a maximum throughput of 3.4 Gbps. The block size of the algorithm is 2^27^ bit ≈ 134 Mbit, and the secrecy parameter we used is 10^−9^ (more details on the extraction are provided in ref. ^23^).

### Protocol description

**Fig. 5:**
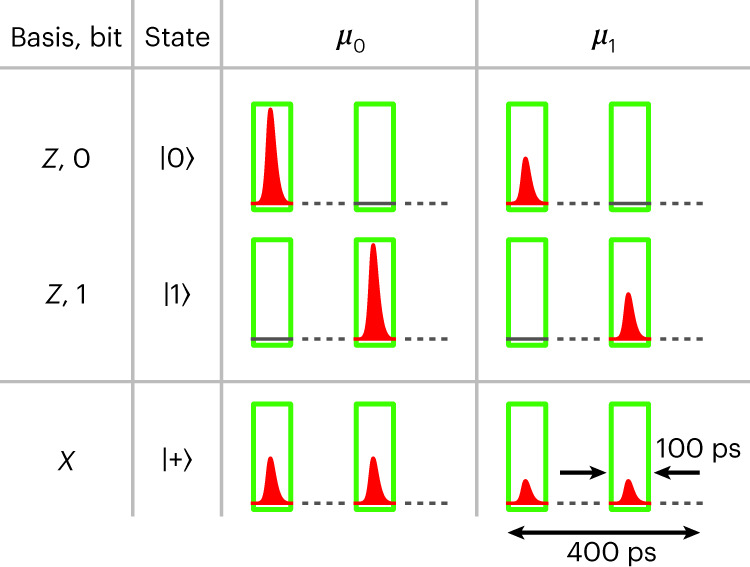
States prepared by Alice. Each state consists of two time bins. The two pulses of one state have a fixed phase relation, but pulses of different states have a random phase relation. Alice chooses the mean photon number *μ*_0_ or *μ*_1_ for each state at random. The green boxes are the detection time windows of Bob, each with a duration of 100 ps.

We use the simplified BB84 with time-bin encoding and one decoy state^13–15^. Alice prepares the states as shown in Fig. 5. In the *Z* basis, we have a pulse either in the early (state ${\left\vert 0\right\rangle}$) or in the late time bin (state ${\left\vert 1\right\rangle}$). State ${\left\vert +\right\rangle}$ of the *X* basis carries pulses in both time bins, but with half the intensity compared to the pulses in the *Z* basis. These two pulses have a fixed phase relation, whereas, between the states, the phase has to be random. Alice chooses the basis at random with probabilities *p*_*Z*, A_ for the *Z* basis and 1 − *p*_*Z*, A_ for the *X* basis. In the case where she chooses the *Z* basis, she picks either ${\left\vert 0\right\rangle}$ or ${\left\vert 1\right\rangle}$ with equal probability. Additionally, she chooses at random between two mean photon numbers *μ*_0_ and *μ*_1_ with probabilities ${p}_{{\mu }_{0}}$ and ${p}_{{\mu }_{1}}$. Bob picks a measurement basis at random with probabilities *p*_*Z*, B_ for the *Z* basis and 1 − *p*_*Z*, B_ for the *X* basis. The secret key is generated from the correlations in the *Z* basis, while the *X* basis is used to find an upper bound on the phase error rate via the decoy method^14^.

### Implementation

A distributed feedback InGaAsP/InP multi-quantum-well laser diode is used to create a train of phase-randomized pulses with FWHM of 45 ps and at a rate of 2.5 GHz. The pulses then pass an imbalanced Michelson interferometer with a time difference of 200 ps between the two arms. The states, as shown in Fig. 5, are encoded using an intensity modulator. The random numbers used to choose the states are produced by AES (Advanced Encryption Standard) cores seeded by a Quantis quantum random number generator (QRNG) from ID Quantique SA. The optimum value of *p*_*Z*, A_ depends on the distance and, in any case, is well above 0.5. As a quantum channel there is an ultra-low-loss (ULL) single-mode fibre. Its dispersion is pre-compensated by dispersion-compensating fibre.

At the other end of the channel, at Bob, the basis is selected passively with the help of a fibre coupler. The optimal probability *p*_*Z*, B_ is close to unity. This means that the sifting efficiency is significantly higher than in the standard BB84. In the *Z* basis, Bob measures the time of arrival of the signal with the multipixel detector described above. In the *X* basis, the pulses pass through a Michelson interferometer with the same delay as that of Alice. Here, the requirement on the detector is less stringent due to the high bias of the basis choice towards the *Z* basis. We chose a MoSi SNSPD with a parallel design (P-SNSPD)^24,25^, which exhibits a timing jitter below 55 ps and a system detection efficiency of 85% at a count rate of 2 Mcps.

Alice uses the FPGA I to control the state preparation. The detections received by Bob are registered by FPGA III. The outputs of the detectors are interfaced to the FPGA III with an in-house-made card. This card can delay the 14 channels of the multipixel detector and the channel of the *X* basis detector individually, allowing us to synchronize them. Furthermore, the card combines the 14 channels of the multipixel detectors into seven channels with OR gates. At very high count rates, the combining of channels will mask some detections. By comparing the count rate of the QKD system with the count rate measured with time-to-digital converters, we found that, due to OR-gate masking, we lose 2.8% of the counts at 320 Mcps.

FPGAs I and III communicate directly via a fibre-optical service channel with a bandwidth of 10 Gbps to synchronize their clocks, for sifting and to perform error correction in real time. During sifting, Bob’s FPGA III sends a short announcement after each detection to Alice’s FGPA I. This contains the time elapsed since the previous detection, the basis and, if the event is the *X* basis, also the bit value of the detected states. No absolute time information is sent by Bob, only the relative time between detections. Alice uses this timing information to identify the corresponding state she sent. She replies by sending Bob the basis used in the state preparation. FPGA I forwards the sifted key to FPGA II, which performs the error correction.

### Key exchange

**Fig. 6:**
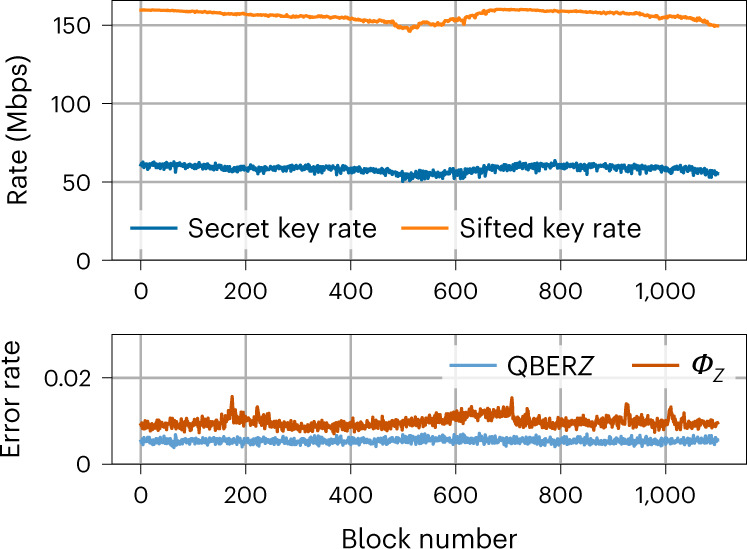
Stability of the key exchange. Measured secret and sifted key rate, QBER in the *Z* basis (QBER *Z*) and phase error rate (*ϕ*_*Z*_) over consecutive privacy amplification blocks of 134 Mbits over 10 km of ULL fibre. The average acquisition time per block was 0.84 s.

We performed secret key exchanges through optical fibres with lengths of 10.0 km and 102.4 km for typically half an hour. In Fig. 6 we see the evolution of the sifted key rate, the SKR, the quantum bit error rate (QBER) *Z* and the phase error rate (*ϕ*_*Z*_) for consecutive privacy amplification blocks. The slight variations of the key rates are due to slow polarization fluctuations and the polarization dependence of the SNSPD quantum efficiency. Note the very low error rates, which suggest that their fluctuations do not impact the SKR significantly. The mean photon number of the signal and decoy states and the probabilities to choose the *Z* basis at Alice and Bob were obtained by numerical optimization for each distance. We managed to exchange secret keys at a rate of 64 Mbps over a distance of 10.0 km and at a rate of 3.0 Mbps over a distance of 102.4 km. Table 1 shows the values of the SKR and the relevant parameters over one privacy amplification block of 134 Mbit (for the two distances).

**Table 1:** Measured SKR and corresponding experimental parameters

Fibre length	Att.	*μ*_0_	*μ*_1_	${p}_{{\mu}_{0}}$	*p*_*Z*, A_	*p*_*Z*, B_	*R*_sift_	*ϕ*_*Z*_	*Q*_*Z*_	SKR
(km)	(dB)						(Mbps)	(%)	(%)	(Mbps)
10.0	1.58	0.49	0.22	0.74	0.65	0.99	159.4	0.8	0.4	64
102.4	16.34	0.46	0.20	0.79	0.66	0.99	7.8	1.0	0.3	3.0

Variables *μ*_0_ and *μ*_1_ are the mean photon number of the signal and decoy states, ${p}_{{\mu}_{0}}$ and ${p}_{{\mu }_{1}} = 1 - {p}_{{\mu}_{0}}$ are the corresponding probabilities to choose these values, *p*_*Z*, A_ and *p*_*Z*, B_ are the probabilities of Alice and Bob to choose the *Z* basis, *R*_sift_ is the sifted key rate, *ϕ*_*Z*_ is the phase error rate and *Q*_*Z*_ is the QBER Z. Att., attenuation.

## Discussion

Although these are best-of-class results, there are still areas in which the QKD set-up could be optimized. Indeed, our QKD scheme was designed to be simple and suitable for a commercial device, and some adaptations could be made to increase even further the maximum SKR.

Due to our high repetition rate and consequently small time bins, we lose the detections that fall outside the time bins (Fig. 2). Whereas further reducing the timing jitter is not simple, we could just double the multipixel detectors at Bob. The advantage would be twofold: the detection rate will be halved on each detector, leading to an almost 10% increase in detection efficiency (Fig. 3), and there will be a decrease in jitter.

Our protocols allow for only one detector in the monitoring basis (projection in only one eigenstate of the *X* basis). To guarantee the security in this configuration we also need to monitor events where Alice used the *Z* basis and Bob measures in the *X* basis, and vice versa. Moreover, we also record events depending on which state was sent previously (some detection depends on two subsequent pulses, see ref. ^14^ for details). This forces us to choose, in the finite key scenario, a lower *p*_*Z*, A_, which lowers the possible achievable sifted key rate.

Finally, the error correction is still not optimal. The used LDPC implementation has a leakage of 17% of the sifted key rate at a QBER of 0.5%. This is much higher than the Shannon limit of 5% of leakage. Rate-adaptive LDPC codes could help minimizing the leakage^26,27^, but corresponding studies do not give information about the leakage at very low QBER. Another solution would be to implement the cascade error correction algorithm^28^, which would allow approaching the Shannon limit, and in fact the state of the art allows for an efficiency of 1.038 and more than 500 MHz of throughput.

Implementing these improvements would allow us to achieve ~140 Mbps at 10 km (under the condition that the other parameters stay the same).

In conclusion, we have demonstrated SKRs up to 64 Mbps over a distance of 10.0 km. This achievement was possible thanks to a QKD system working at a high repetition rate of 2.5 GHz, coupled with our custom SNSPDs and readout electronics, which allow us to detect with low jitter and high efficiency at a high count rate. This result paves the way for secret key-demanding applications like real-time one-time-pad secured video encryption in a metropolitan area.

## Online content

Any methods, additional references, Nature Portfolio reporting summaries, source data, extended data, supplementary information, acknowledgements, peer review information; details of author contributions and competing interests; and statements of data and code availability are available at 10.1038/s41566-023-01168-2.

## Data Availability

The data that support the findings of this study are available from the corresponding author upon reasonable request.

## Code availability

The computer code that supports the findings of this study is available from the corresponding author upon reasonable request.
